# Supplementary material for: Serum Proteomics in Patients with Head and Neck Cancer: Peripheral Blood Immune Response to Treatment
Source: Int J Mol Sci. 2022 Jun 4;23(11):6304. doi: 10.3390/ijms23116304 (PMC9180944; doi:10.3390/ijms23116304)
Supplement: Supplementary file 1 [file ijms-23-06304-s001.zip › ijms-1738923-Supplementary Table S1.pdf]

Table S1. Proteins analyzed (n = 83) with multiplex immuoassay in patients with head and neck cancer.

IL-1 alfa<br>IL-2<br>IL-4<br>IL-5<br>IL-6<br>IL-7<br>IL-8<br>IL-10<br>IL-12<br>IL-12RB1<br>IL-13<br>IL-18<br>IL-21<br>IL-33<br>IL-35	CD4<br>CD5<br>CD8A<br>CD27<br>CD28<br>CD40<br>CD40-L<br>CD70<br>CD83<br>CD244	ADA<br>ADGRG1<br>ANGPT1<br>ANGPT2<br>ARG1<br>CAIX<br>CASP-8<br>CRTAM<br>DCN<br>FASLG<br>Gal-1<br>Gal-9<br>GZMA<br>GZMB	HO-1<br>ICOSLG<br>KLRD1<br>LAMP3<br>LAP<br>TGF-<br>beta<br>MIC-<br>A/B<br>NCR1<br>NOS3<br>PD-L1<br>PD-L2<br>PTN<br>TIE2<br>TRAIL<br>TWEAK
TNF<br>TNFRSF4<br>TNFRSF9<br>TNFRSF14<br>TNFRSF21<br>TNFSF14	CCL3<br>CCL4<br>CCL17<br>CCL19<br>CCL20<br>CCL23	CXCL1<br>CXCL5<br>CXCL9<br>CXCL10<br>CXCL11<br>CXCL12<br>CXCL13<br>CX3CL1	
PDGF subunit 8<br>PDCD1	IFN-beta<br>IFN-gamma	MMP7<br>MMP12	MCP-1<br>MCP-2<br>MCP-3<br>MCP-4
